# Disruptions in Sexual and Reproductive Health Care Service Delivery for Immigrants During COVID-19

**DOI:** 10.1089/whr.2023.0004

**Published:** 2023-07-05

**Authors:** Goleen Samari, Heather M. Wurtz, Mihiri Karunaratne, Kate Coleman-Minahan

**Affiliations:** ^1^ Heilbrunn Department of Population and Family Health, Mailman School of Public Health, Columbia University, New York, New York, USA.; ^2^ Anthropology Department, University of Connecticut, Storrs, Connecticut, USA.; ^3^ Research Program on Global Health and Human Rights, Human Rights Institute, University of Connecticut, Storrs, Connecticut, USA.; ^4^ Population Studies and Training Center, Brown University, Providence, Rhode Island, USA.; ^5^ School of Medicine, University of California, San Diego, La Jolla, California, USA.; ^6^ College of Nursing, University of Colorado Anschutz Medical Campus, Aurora, Colorado, USA.

**Keywords:** sexual and reproductive health, immigrants, care delivery, COVID-19

## Abstract

**Purpose:**

To provide perspectives from heterogenous cisgender immigrant women and service providers for immigrants in New York City (NYC) on how restrictive sexual and reproductive health (SRH) care delivery environments during COVID-19 shape immigrant's access to health care and health outcomes to generate insights for clinical practices and policies for immigrant women's health care needs.

**Methods:**

A qualitative study was conducted in 2020 and 2021, including in-depth interviews with 44 immigrant women from different national origins and 19 direct service providers for immigrant communities in NYC to explore how immigrants adapted to and were impacted by pandemic-related SRH care service delivery barriers. Interviews were coded and analyzed using a constant comparative approach.

**Results:**

Pandemic-related delays and interrupted health care, restrictive accompaniment policies, and the transition from in-person to virtual care compounded barriers to care for immigrant communities. Care delays and interruptions forced some participants to live with untreated health conditions, resulting in physical pain and emotional distress. Participants also experienced challenges within the health care system because of changes to visitor policies that restricted the accompaniment of family members or support persons. Some participants experienced difficulties accessing telehealth and technology, while others welcomed the flexibility given the demands of frontline work and childcare.

**Conclusions:**

To mitigate the health and social implications of increasingly restrictive immigration, reproductive, and social policies, clinical practices like expanding access to care for all immigrants, engaging immigrant communities in health care institutions policies and practices, and integrating immigrant's support networks into care play an important role.

## Introduction

More than 44 million immigrants and 8 million citizens with at least one undocumented family member live in the United States.^1,2^ Immigrants account for 17% of cisgender women of reproductive age and there is considerable heterogeneity by national origin. Before the COVID-19 pandemic, women in immigrant and minoritized communities had greater barriers accessing sexual and reproductive health (SRH) care,^3,4^ reported poorer quality of care,^5,6^ and experienced poorer SRH outcomes than the US-born population.^4^ The limited existing literature^7^ on immigrants' SRH primarily focuses on prenatal care and gynecological cancer screening practices, with less of a focus on SRH experiences and fertility, contraception, and abortion.^8^ Furthermore, while immigrant populations face well-known social, economic, and political barriers to obtaining SRH care, these barriers stem from intersecting consequences of structural inequalities, xenophobic immigration policy, and racism,^9–12^ which are rarely the focus of immigration SRH research.^3^

COVID-19 offers a glimpse into the consequences of structural barriers for immigrant access to SRH care. SRH care is an important emphasis of study, given the pervasive impact of oppressive structures and restrictive policy making that leave SRH care uniquely inaccessible and stigmatized relative to other aspects of health and health care.^13,14^ During COVID-19, SRH services, which are essential primary care, were negatively impacted by shifting priorities within health care systems.^13,15^

Studies with service providers demonstrated the scope of tremendous interruptions to SRH services through shifts from in-person to virtual visits, supply chain disruptions, and reduced staffing.^15^ Access to SRH care was further reduced by loss of health insurance and wages due to pandemic-related job losses and fears of exposure to the virus.^3,16^ There were also lower rates of sexually transmitted infection screening, reduced access to contraception and abortion care, and reduced access to services for intimate partner violence (IPV).^17,18^ Yet little is known about how these changes in SRH service delivery during COVID-19 were experienced among immigrant communities.^19^

To fill this gap in knowledge among a large, underserved population in the United States, we conducted a qualitative study among a unique sample that includes both cisgender immigrant women from diverse national origins and direct service providers for immigrant communities in New York City (NYC). We use the perspectives of immigrant women and direct service providers to describe immigrant women's experiences of disruptions in SRH care delivery during COVID-19 and discuss clinical and policy implications.

## Methods

A qualitative study, including semistructured, in-depth interviews, was conducted with cisgender immigrant women in NYC from several regions and countries of origin and direct service providers who serve immigrant communities from all five boroughs (Bronx, Brooklyn, Manhattan, Staten Island, and Queens). Interviews explored experiences of SRH care services among women and providers during the first and second years of the COVID-19 pandemic (September 2020–May 2021). Institutional Review Board (IRB) approval was received for the study (AAT2404). Table 1 includes eligibility criteria for both samples.

**Table 1.** Sample Eligibility Criteria (Immigrants and Direct Service Providers)

Immigrant women	Direct service providers
***n*** = 44	***n*** = 19
18–49 years old	Serves in a leadership role
Cisgender woman	Works for an immigrant serving organization^a^
Born outside the United States or to at least one parent born outside the United States	Works for a CBO, family planning clinic, social services agency, or health facility
Lives in a NYC borough	Provides services in a NYC borough
Speaks English or Spanish	Speaks English or Spanish

^a^ Client populations served ranged from Latinx, Asian, Arab, South Asian, Black, and all immigrant communities.

NYC, New York City.

Recruitment of the immigrant sample began in August 2020 and interviews were completed by March 2021. Community-based organizations (CBOs) that work with and provide services (health, education, social work, gender-based violence, legal, and financial) for immigrant communities facilitated recruitment by identifying participants, posting flyers, and distributing emails to their clients. Recruitment for direct service providers began in March of 2021 and concluded in May of 2021. The CBOs that facilitated recruitment for immigrant women also facilitated initial outreach for service provider interviews, and additional service provider participants were recruited through snowball sampling.

The interview guides were developed by the study team based on the literature on immigrants' SRH and access to health care and revised and finalized after initial interviews. Interviews were conducted in either English or Spanish, depending on participant preference, through Zoom video or phone calls per participant preference. Spanish interviews were conducted by one of the bilingual investigators on the research team. Interviews lasted 45–60 minutes and were audio recorded. Each participant received an information sheet about the study and provided verbal consent for participation and audio recording before the interview. Participants were given a $50 Amazon gift card for their participation in the study.

Recordings in English were transcribed using transcription software and transcripts were manually compared with the audio recording for accuracy. A certified transcriber and certified translator transcribed and translated the Spanish interviews to English. We utilized separate coding and analytic processes for each sample. Codebooks were developed with a subset of each sample's transcripts using a line-by-line approach to identify prominent themes and patterns. Thematic codes based on existing literature were also integrated into the codebooks. Several rounds of codebook revisions were conducted until there was a high level of agreement among the study team about the meaning and application of codes.

Transcripts were deidentified, uploaded, and coded using *Dedoose*. Data for both samples were analyzed using a constant comparative method,^20^ which is an iterative process of continuously reviewing data alongside emergent themes and patterns until core categories and relationships between categories are identified. The study investigators developed an initial codebook that was revised, finalized, and applied with a four-member study team through an iterative coding and meeting process to review and resolve codes, questions, and discrepancies. This helped ensure validity and consistency of coding across team members and identification of all possible themes. Once all transcripts were coded, we identified and described themes and narratives that emerged around immigrant women's and direct service providers' experiences with SRH care service delivery during COVID-19. Common themes across the two samples were combined using detailed memos and study team meetings to generate cross-sample thematic results.

## Results

The sample includes 44 immigrant women and 19 direct service providers. Sample characteristics are provided in Tables 2 and 3. Immigrant participants had a mean age of 33 years; 49% were from Latin America and 71% were undocumented. About 42% of immigrant women were married or cohabiting and 54% were single or separated. The 19 direct service providers represented leadership and clinicians at CBOs (63%), family planning clinics (16%), and family practices or community health clinics (22%) across all five boroughs.

**Table 2.** Descriptive Characteristics (Means or %) for Immigrant Women

Key variables	Immigrant women (***n*** = 44)	
	***n***	% or mean (SD)
Current age (years)	44	33.40 (7.26)
Region of origin		
Latin America	22	50.0
South Asia	11	25.0
East Asia	6	13.6
Middle East and North Africa	5	11.4
Marital status		
Married	10	22.7
Cohabiting	9	20.5
Single	11	25.0
Separated/divorced/widow	14	31.8
Residence		
Bronx	9	20.5
Manhattan	12	27.3
Queens	18	40.9
Brooklyn	6	13.6
Years in the United States	44	14.23 (9.66)
Documented	31	70.5
Employed	27	61.4
Have a child	23	52.2
Spanish interview	16	36.4

SD, standard deviation.

**Table 3.** Descriptive Characteristics of Direct Service Providers

Key variables	Direct service providers (***n*** = 19)	
	***n***	%
Type of organization		
Community based	12	63
Health care clinic	4	21
Family planning clinic	3	16
Borough		
Bronx	2	11
Brooklyn	2	11
Manhattan	11	58
Staten Island	2	11
Queens	2	11
Service type		
Gender-based violence	5	26
Immigration/social work	7	37
Sexual and reproductive health^a^	7	37

^a^ Three of the seven SRH service providers also provided primary care.

SRH, sexual and reproductive health.

### SRH needs and vulnerabilities

Participants explained how they contended with a range of SRH concerns over the course of the pandemic. When asked if they experienced any change in their SRH during the COVID-19, 12 (27%) women reported changes in their menstruation, including early and late onset of periods, missed periods, irregular bleeding, and increased menstrual pain. Eight women (18%) reported other ailments, including vaginal yeast infections, pain during intercourse, and obstetric complications. And others were receiving care for problems diagnosed before the pandemic, including thyroid cancer, breast cysts, uterine fibroids, and an abnormal cervical cancer screening.

Fifteen participants reported managing current chronic illnesses (*e.g.,* asthma, metabolic disorders) and eight reported a history of IPV. Most participants reported experiencing mental health distress, such as sadness or anxiety, and some described mental health illnesses, including depression, substance use disorders, and eating disorders. Below, we describe the main SRH service delivery barriers that emerged for immigrants and providers, including the consequences of delayed and interrupted health care, harm from restrictive accompaniment policies, and mixed experiences with transition to telehealth. Table 4 includes illustrative quotes from the participants for each theme.

**Table 4.** Themes and Quotations from Immigrant Women and Direct Service Providers for Immigrant Communities in New York City (*n* = 63)

**Consequences of cancelled and delayed health care** “I'm hearing a lot of comments about, you know, *‘I've had these appointments canceled that I had been waiting for, for a very long time*’…” (Physician, Family Medicine Clinic, Bronx). “I went there. Just… just for my regular checkup and everything, but they canceled on me and I went, but they didn't, they didn't send me any notification that it had just got cancel. So I had to go there and find out that everything was closed. And, yeah, I just came home” (Immigrant woman, 23 years old, Bangladesh). “She (patient who presented with a painful lump in her breast) doesn't have citizenship papers and so had established care at a public clinic…still it's been such a struggle to get her in for those appointments and like we [medical staff] have called ourselves. Right? So, we tried to, like, flex our own privilege and see if we could get these appointments and we can't really get through…” (Physician, Family Medicine Clinic, Manhattan). “…we weren't sure how to have them quickly access like long acting, reversible contraception. So if they want an IUD, like how can we schedule you within, like, a clinic, like the restricted schedule?” (Physician, Family Medicine Clinic, Manhattan). “I had [a PAP smear] scheduled for April. They sent me a text message to tell me that the appointments were canceled and that they would get back to me as soon as there was one and to this day, they have not called me. I called the other day, but it just went to voicemail; there is no way to make appointments” (Immigrant woman, 45 years old, Mexico). “Unfortunately, you know, again, if you're undocumented, there are… Of course, immigrants doesn't mean everybody is undocumented. But even when the client has insurance, there are long waits. There's waiting lists for client [mental health] services. And that was even pre-COVID” (Nurse Practitioner, Community Health Clinic, Bronx).
**Harm from restrictive accompaniment policies** “Child care is a big, big issue, especially because early in the pandemic, we didn't want people coming with, like we wanted people coming alone. And so people with children would miss appointments” (Physician, Family Medicine Clinic, Manhattan). “[My friend] was sick, so sick, but she didn't go, she didn't go anywhere because her kids are home…she didn't have any support for someone to come and watch the kids” (Immigrant woman, 21 years old, Yemen). “When the pandemic started, I thought: I am going to die, I will never see them, I am medically fragile, what would happen if I have to be isolated, what would happen to my daughter, who would I leave her with, so many worries” (Immigrant woman, 39 years old, Dominican Republic). “[The uterine cysts] got so bad…They have to then go in and have surgery and take it out and all that stuff. But [my mother] just didn't feel comfortable doing that [procedure] alone” (Immigrant woman, 24 years old, Bangladesh). “…you can imagine somebody who's not fluent in English, who needs an interpreter, who needs that family member as a support. I mean, anybody needs family members during the delivery. But I think it's even more so compounded by the fact that that our immigrant populations really depend on that close family support to navigate in this country. And yet we were telling them, you can't have anybody with you during something as scary as like an urgent C-section…” (Physical, Family Planning Clinic, Queens).
**Mixed experiences with telehealth** “Sometimes the internet cuts out, it is a little tiring because you're talking like 30 minutes, 40 minutes and suddenly poof! It turns off, and re-connects and again poof! it turns off and you have to do it by phone, you know the internet sometimes fails, so it is a bit frustrating… (Immigrant woman, 31 years old, Mexico*).* “When the COVID started, right, our lives literally shifted to a virtual world, right? Now, there's no, there's no in-person contact… our [clients], they have limited literacy when it comes to English, when it comes to technology. So, I know it's going to be a challenge for us to deliver the workshops, to deliver our message to them virtually because we would literally have to…train them to use Zoom” (Immigrant woman, 23 years old, Bangladesh). “I didn't like it because how is he going to know without looking at my body, without touching me, without drawing blood? How is he going to know… you know? So I was always in doubt, because the doctor only looked at my face, he didn't look at my body, how does he know that's what it was?” (Immigrant woman, 38 years old, Honduras). “…my patients who work, especially like people who became pregnant…the fact that we were doing prenatal visits on a [hybrid] schedule…had people take less time off of their work, which allowed them to get health care without using those days or without feeling like this fear of like, *‘oh, they could replace me or fire me if I take too much time off…*’ (Physician, Family Planning Clinic, Manhattan). “Then there was this big push from national organizations and from our own hospital, our own department, about being able to safely provide obstetrical care using just video… Which one may think, “well, you're not listening to my baby's heartbeat, you're not examining my belly. How can you guarantee that my baby is safe?” And the evidence, quote unquote, at that point is that, “no, if you if the baby's moving, then everything's fine,” which I understand the leadership position and try to minimize the risk of COVID but I've heard so many women saying, *“I feel unsafe. I don't think this is optimal care”*” (Physician, Family Planning Clinic, Bronx). “The biggest challenge was that they could not physically come in. Right. And so we switched to help, with telehealth. Almost overnight and. I mean, I think telehealth has a place, but I also think it has a lot of barriers, and especially for immigrant women who maybe don't have access to Internet, don't have the right equipment, you know, there might be using just some old crappy phone. It's hard to get on. There's a knowledge issue on how to log on. There's a there's a language barrier. There is. I mean, there was just it was really, really hard for women to access care” (Physician, Community Health Clinic, Manhattan). “…things that go untreated like STDs that maybe won't get diagnosed, I mean. Usually, yes, people will come in with a symptom we screen. They have like chlamydia, whatever, but we usually find it upon a routine screening. So if you're not coming in for your screenings, then we're missing a lot of STDs in that capacity. Yes, that, I think [was] a big issue [during the pandemic]…under the best of circumstances, that's an issue” (Nurse Practitioner, Family Planning Clinic, Manhattan).

IUD, intrauterine contraceptive device; PAP, papanicolaou test; STD, sexually transmitted disease.

### Consequences of delayed and interrupted health care

Respondents discussed the negative impact of cancelled and delayed health care appointments on women's SRH during the pandemic, including decreased access to family planning services and delayed chronic illness management. Respondents noted challenges for immigrant women in need of services, which required in-person clinic visits, such as the insertion or removal of long-acting forms of contraception (*e.g.,* intrauterine devices) and abortion care. Some providers described how abortion was still accessible, but often delayed to later in pregnancy.

Cancelled and delayed appointments took a toll on immigrant women with complicated SRH conditions that required ongoing management. Barriers that are specific to immigrant communities like lack of health insurance, not having a regular source of primary care, low knowledge about and distrust in the health care system, reduced care continuity, fewer opportunities for culturally concordant care, and limited assistance in languages besides English were exacerbated during COVID-19, prolonging what had already been extensive waiting periods for women to receive treatment and care.

Care was also interrupted when providers discharged hospitalized patients earlier than the standard of care, to reduce COVID-19 exposure and free up hospital beds. One physician, for instance, noted that many of his pregnant immigrant patients during the pandemic felt “they were like pushed out early from the hospital,” which providers felt would shape trust and future engagement in health care for immigrant mothers and their children.

Care delays and interruptions forced some immigrant women to live with untreated health conditions, resulting in physical pain and mental health distress. One participant (*38 years old, Ecuador)* had experienced a recurrence of thyroid cancer; when the pandemic started, her radiation therapy came to an abrupt halt. Another woman *(35 years old, Ecuador)* had to postpone surgery to remove uterine fibroids, resulting in additional months of heavy bleeding, while she was juggling securing work and her childcare needs.

Immigrant women described having very limited social support to help address the physical, emotional, and logistical needs (*e.g.,* help with childcare) associated with delayed care and untreated conditions because they did not have family in the United States. One woman (26 years old, Yemen), for example, who had recently given birth, had to wait 6 months for an operation to repair an obstetric fistula after the surgeon was diagnosed with COVID-19.

While awaiting surgery, she had to undergo biweekly hospital visits and endured tremendous physical pain, while also taking care of her newborn baby. During this time, her husband was still in Yemen because of travel and immigration restrictions implemented by the Trump administration. Her father had passed away from COVID-19, and her mother had stayed in Yemen following the funeral. She reflected on the difficulties of delayed care, given this lack of support: “It was very hard. I had my moments where I wake up the middle of the night and I just start crying [so hard that] I can't even…And it's just like, who should I ask right now? Who [can] help me?.”

### Harm from restrictive accompaniment policies

Women also faced challenges within the health care system because of changes to visitor policies that restricted the accompaniment of family members or support persons. Women were deterred from seeking care because they could no longer count on the support of family members, especially their children, during clinical visits to help overcome language barriers and manage other forms of cultural brokerage.

One participant *(24 years old, Bangladesh)* described how her mother put off a necessary surgery to remove gynecologic cysts because of the fear of going through the procedure alone. Another participant (*34 years old, Mexico*) was so afraid to seek care without the support of a family member that she waited until her daughter's dental appointment to ask for professional assistance for what was later diagnosed as extreme anxiety. She said that she was “embarrassed to talk to people,” so she told her daughter, “You speak to them in English and I'll tell you what to say…”

Some women were forced to forgo medical appointments because visitor policies prohibited them from bringing their children and they had limited or no childcare. Out of the 24 women with children in our sample, 15 (63%) were separated, divorced, or single and the primary caretaker of their children and even those in partnerships were often the primary caretaker and worked to support the family.

Many participants expressed a profound fear of being isolated and separated from their children if they or their children were to require hospitalization. For some, this fear informed their decisions to forego care when they themselves or their children were ill, even if they thought it was necessary. One participant (*39 years old, Mexico),* for instance, described the difficult decision she was forced to grapple with when both of her children became ill in the early months of the pandemic. “I thought that if I went [to the hospital], they would possibly isolate them from me because they would not allow any family members to be there. I panicked, I was afraid, I decided to leave them at home.”

### Mixed experiences with telehealth

When care was not delayed or interrupted, it was often switched to virtual or phone visits, which came with both challenges and benefits. The switch to telehealth exacerbated digital divides, including access to the internet and technological resources. Some immigrant women experienced difficulties accessing technology, including consistent, high-quality internet connectivity. For other women, their lack of familiarity with technology and different kinds of virtual platforms paired with limited English proficiency, created a substantial learning curve to engage with virtual health care services.

The availability of in-person appointments emerged as critical for allowing women to report concerns that they might feel afraid or ashamed of revealing—or that they may not even be fully aware of—and have difficultly broaching through by phone or video and with household members present, such as mental health challenges or IPV. Participants and providers frequently discussed under-reporting of IPV during COVID-19 as participants were trapped at home with their aggressors and could not safely discuss IPV-related issues through a virtual visit.

A social worker at a Family Planning clinic in Manhattan commented, “…even reporting is unsafe because it's like where do you find a safe place to have that conversation at home?” Providers also shared concerns about privacy noting that women “don't have a lot of space in their homes. Right? They night be living in one or two rooms with their children who are remotely going to school and maybe their mother-in-law, who is in the other room within hearing distance. And so the kinds of questions that we ask in reproductive health are really personal. And I just found that was really unfair.”

Women and providers shared concerns about whether the quality of virtual care was like in-person care. One woman (*38 years old, Honduras*), for example, attended a virtual consultation with a physician after her menstrual cycle was delayed for over 2 months. Even though she was diagnosed with a menstrual disorder, she continued to be concerned with her cycle. When asked about her experience with the telehealth appointment, she replied: “I was always in doubt, because the doctor only looked at my face, he didn't look at my body, how does he know that's what it was?”

Although many women found virtual appointments challenging, some women reported positive experiences, particularly when it came to counseling for mental health and other sources of psychosocial support. Many found a deep sense of relief in being able to begin or continue counseling without risking exposure to COVID-19. Some service providers relayed those virtual appointments increased access to care for women who had other constraints, such as full-time jobs with limited time flexibility. One health care provider commented that offering prenatal visits both in-person and virtually allowed women to access care without having to opt for unpaid time off or “without feeling like this fear of like, ‘oh, they could replace me or fire me if I take too much time off.…”

## Discussion

Although health care was delayed and interrupted for almost everyone during the COVID-19 pandemic, in this qualitative study with a large, heterogenous sample of immigrant women and service providers, we found that pandemic-related delayed and interrupted health care, restrictive accompaniment policies, and the transition from in-person to virtual care compounded barriers to care faced by immigrant women before the pandemic. Data illustrate that immigrant women experienced physical and emotional suffering during COVID-19 related to SRH service delivery because their social status as immigrants was a compounded vulnerability as an unprepared and under-resourced health care system attempted to adapt. Changes at the policy and health care system levels are needed to increase health equity for immigrant communities.

This study describes ways that immigrant women's health care suffered during the pandemic. Cancelled health care appointments were common at the pandemic's onset due to risk of exposure and reduced staffing.^21,22^ Our findings align with existing literature that COVID-related cancellations compounded already substantial barriers to care and resulted in unnecessary progression of health conditions and mental distress for uninsured or underinsured immigrant women,^23,24^ and further show that delays in SRH care are an added dimension of care discontinuity for immigrants during the pandemic. For under-resourced communities, finding timely and adequate care is often hampered by bureaucratic hurdles, complicated financial assistance plans, and a lack of care continuity.^25–27^

Findings demonstrate how immigrant women's already limited access to specialty care and management of a range of health conditions, SRH related and otherwise, caused physical and mental distress as they were denied essential health care, including obstetric surgeries, cancer treatment, and other chronic illness management. Because chronic illness management requires access to care,^28,29^ study findings confirm that lack of health care access for immigrants is associated with reduced confidence in the health care system and future engagement.^24,30^ Clinical efforts are needed to close gaps in equitable provision of care, including supporting primary care providers to help uninsured patients access to specialty care. Health care institutions can engage community members and local governments in planning and reassessment of the definition of “essential care,” and create systems to ensure cancelled appointments are rescheduled quickly.

Study findings highlight how the unique sociopolitical and health care policy environment for immigrants intensified the negative experiences related to COVID-19 restrictions on accompaniment policies. Advancing research that found accompaniment policy restrictions reduced the ability of patients' family members to help navigate health care institutions, leading to negative patient experiences and worse health outcomes^31^; we found that restricting patients from bringing support people to health care visits was consequential for health care access for immigrants through exacerbating language barriers, lack of familiarity with the health care system, and mistrust of the health care system. Language and communication barriers exacerbate immigrants' experiences of discrimination in health care settings.^32,33^

Even when formal interpreters are available, support persons are crucial to creating safe spaces for immigrant women to navigate the health care system and receive needed emotional support, cultural brokerage, and added trust.^34,35^ Moreover, given the sensitive issues involved in SRH care for immigrant women, support persons may play a larger-than-anticipated role in access to care, care delivery, and quality of care, and should be integrated into care, while protecting patient privacy and autonomy. Health care institutions should welcome and integrate support persons in primary care, including SRH care, and should engage patients and communities when designing or changing accompaniment policies to ensure patients' needs are met, while addressing public health and institutional priorities.

Participants' experiences with virtual SRH services provide insight for addressing structural obstacles and refining best practices now that in-person and hybrid services are offered more consistently. Virtual care can expand or expedite access for immigrant and other underserved communities and should be offered to everyone, particularly those who express concerns with taking time-off from work, childcare, transportation, and in other resource-limited situations. Simultaneously, efforts should also be made in virtual encounters to connect patients with social services to provide patients a full scope of options. In keeping with current guidance, when determining the appropriateness of virtual care, providers should consider their existing relationship with patients, if any, as well as patients' preferences and barriers, including internet access and privacy.^36^

Furthermore, virtual visits should still be consistent with standards of care, including providing mental health screening, IPV screening, and vaccine counseling. Issues of digital redlining and digital fluency, which impact marginalized populations, particularly immigrant women,^37,38^ require structural interventions, such as discounts for internet and cellular services, equity-focused technology monitoring programs, and expansion of broadband connectivity.^39^ In the meantime, health care staff should assess barriers to virtual care before visits and offer resources or work directly with patient's support networks as participants identified support networks as helpful in overcoming technological barriers.

Finally, study findings show how primary care is both an essential source of SRH care and is an important entry point to care for immigrant women. As reproductive health care, specifically abortion and contraception, are restricted in the United States, primary care providers will become even more of an essential source of SRH care and an entry point to care.

Although women may seek out SRH care for SRH reasons (*e.g.,* STI screening, contraception), these visits provide an opportunity for women to discuss a range of health and social concerns and for providers to assess other health and social needs, including safety, therefore serving as a principal entry point for connecting women to primary and specialty care, mental health, and other social resources. As evidenced by our results, immigrant women may use their children's health care visits to access their own care, and others described their fear of being separated from their children during health care encounters. Study data further confirm the importance of both primary care and SRH care to fully support the comprehensive needs of immigrant women and their families.^40^

The study was conducted in NYC and generalizability to health care systems and populations elsewhere is limited. However, there is a critical lack of data on health care behaviors among immigrant communities, particularly those who lack documentation. We highlight important considerations for health care settings that serve immigrant populations, including expanding access to care for all immigrants, engaging immigrant communities in health care institutions policies and practices, and integrating immigrant's support networks into care. More research about how to deliver health care, whether SRH care specifically or primary care, is needed to mitigate the health and social implications of increasingly restrictive immigration, reproductive, and social policies.

## Abbreviations Used

CBOs: community-based organizations
IPV: intimate partner violence
IRB: institutional review board
IUD: intrauterine contraceptive device
NYC: new york city
PAP: papanicolaou test
SD: standard deviation
SRH: sexual and reproductive health
STD: sexually transmitted disease

## References

[1] Esterline C, Batalova J. Frequently Requested Statistics on Immigrants and Immigration in the United States [Internet]. migrationpolicy.org. 2022. Available from: https://www.migrationpolicy.org/article/frequently-requested-statistics-immigrants-and-immigration-united-states [Last accessed: March 15, 2023].

[2] Mathema S. Keeping Families Together: Why All Americans Should Care About What Happens to Unauthorized Immigrants. Center for American Progress: Washington, DC.; 2021.

[3] Desai S, Samari G. COVID-19 and immigrants' access to sexual and reproductive health services in the United States. Perspect Sex Repro H 2020;52(2):69–73. 10.1363/psrh.12150 PMC7307065 32530526

[4] Tapales A, Douglas-Hall A, Whitehead H. The sexual and reproductive health of foreign-born women in the United States. Contraception 2018;98(1):47–51. 29453946 10.1016/j.contraception.2018.02.003 PMC6029875

[5] Ornelas IJ, Yamanis TJ, Ruiz RA. The health of undocumented Latinx immigrants: What we know and future directions. Ann Rev Public Health 2020;41:289. 32237989 10.1146/annurev-publhealth-040119-094211 PMC9246400

[6] Pitkin Derose K, Bahney BW, Lurie N, et al. Immigrants and health care access, quality, and cost. Med Care Res Rev 2009;66(4):355–408. 19179539 10.1177/1077558708330425

[7] Tapales A, Desai S, Leong E. Data opportunities for studying the sexual and reproductive health of immigrants in the United States. J Health Care Poor Underserved 2019;30(2):560–586. 31130538 10.1353/hpu.2019.0031 PMC6691731

[8] Jain T, LaHote J, Samari G, et al. Publicly-funded services providing sexual, reproductive, and maternal healthcare to immigrant women in the United States: A systematic review. J Immigrant Minority Health 2022;24(3):759–778. 10.1007/s10903-021-01289-2 PMC10373793 34697702

[9] Derose KP, Escarce JJ, Lurie N. Immigrants and health care: Sources of vulnerability. Health Affairs 2007;26(5):1258–1268. 17848435 10.1377/hlthaff.26.5.1258

[10] Viruell-Fuentes EA, Miranda PY, Abdulrahim S. More than culture: Structural racism, intersectionality theory, and immigrant health. Soc Sci Med 2012;75(12):2099–2106. 22386617 10.1016/j.socscimed.2011.12.037

[11] Samari G, Nagle A, Coleman-Minahan K. Measuring structural xenophobia: US State immigration policy climates over ten years. SSM Population Health 2021;16:100938. 34660879 10.1016/j.ssmph.2021.100938 PMC8503659

[12] Misra S, Kwon SC, Abraído-Lanza AF, et al. Structural racism and immigrant health in the United States. Health Educ Behav 2021;48(3):332–341. 34080482 10.1177/10901981211010676 PMC8935952

[13] Hall KS, Samari G, Garbers S, et al. Centring sexual and reproductive health and justice in the global COVID-19 response. Lancet 2020;395(10231):1175–1177. 32278371 10.1016/S0140-6736(20)30801-1 PMC7146687

[14] Riley T, Zia Y, Samari G, et al. Abortion criminalization: A public health crisis rooted in white supremacy. Am J Public Health 2022;112(11):1662–1667. 36223577 10.2105/AJPH.2022.307014 PMC9558193

[15] Maier M, Samari G, Ostrowski J, et al. ‘Scrambling to figure out what to do’: A mixed method analysis of COVID-19's impact on sexual and reproductive health and rights in the United States. BMJ Sex Reprod Health 2021;47(4):e16–e16. 10.1136/bmjsrh-2021-201081 PMC8359865 34376524

[16] Đoàn LN, Chong SK, Misra S, et al. Immigrant communities and COVID-19: Strengthening the public health response. Am J Public Health 2021;111(S3):S224–S231. 34709878 10.2105/AJPH.2021.306433 PMC8561064

[17] Lindberg LD, Mueller J, Kirstein M, et al. The continuing impacts of the COVID-19 pandemic in the United States: Findings from the 2021 Guttmacher survey of reproductive health experiences. 2021. Available from: https://www.guttmacher.org/report/continuing-impacts-covid-19-pandemic-findings-2021-guttmacher-survey-reproductive-health [Last accessed: March 15, 2023].

[18] Lindberg LD, VandeVusse A, et al. Early Impacts of the COVID-19 Pandemic: Findings from the 2020 Guttmacher Survey of Reproductive Health Experiences. 2020. Available from: https://www.guttmacher.org/report/early-impacts-covid-19-pandemic-findings-2020-guttmacher-survey-reproductive-health [Last accessed: March 15, 2023].

[19] Mukherjee TI, Khan AG, Dasgupta A, et al. Reproductive justice in the time of COVID-19: A systematic review of the indirect impacts of COVID-19 on sexual and reproductive health. Reprod Health 2021;18(1):252. 34930318 10.1186/s12978-021-01286-6 PMC8686348

[20] Glaser BG. The constant comparative method of qualitative analysis. Soc Problems 1965;12(4):436–445.

[21] Moynihan R, Sanders S, Michaleff ZA, et al. Impact of COVID-19 pandemic on utilisation of healthcare services: A systematic review. BMJ Open 2021;11(3):e045343. 10.1136/bmjopen-2020-045343 PMC7969768 33727273

[22] Birkmeyer JD, Barnato A, Birkmeyer N, et al. The impact of The COVID-19 pandemic on hospital admissions in the United States. Health Affairs 2020;39(11):2010–2017. 32970495 10.1377/hlthaff.2020.00980 PMC7769002

[23] Clark E, Fredricks K, Woc-Colburn L, et al. Disproportionate impact of the COVID-19 pandemic on immigrant communities in the United States. PLoS Negl Trop Dis 2020;14(7):e0008484. 32658925 10.1371/journal.pntd.0008484 PMC7357736

[24] Ross J, Diaz CM, Starrels JL. The disproportionate burden of COVID-19 for immigrants in the Bronx, New York. JAMA Intern Med 2020;180(8):1043. 32383754 10.1001/jamainternmed.2020.2131

[25] Luque JS, Soulen G, Davila CB, et al. Access to health care for uninsured Latina immigrants in South Carolina. BMC Health Serv Res 2018;18(1):310. 29716586 10.1186/s12913-018-3138-2 PMC5930513

[26] Lee AA, James AS, Hunleth JM. Waiting for care: Chronic illness and health system uncertainties in the United States. Soc Sci Med 2020;264:113296. 32866715 10.1016/j.socscimed.2020.113296 PMC7435333

[27] Roder-DeWan S, Baril N, Belanoff CM, et al. Being known: A grounded theory study of the meaning of quality maternity care to people of color in Boston. J Midwifery Womens Health 2021;66(4):452–458. 34240539 10.1111/jmwh.13240 PMC8456935

[28] Chin MH. Uncomfortable truths—What Covid-19 has revealed about chronic-disease care in America. N Engl J Med 2021;385(18):1633–1636. 34677919 10.1056/NEJMp2112063

[29] Kendzerska T, Zhu DT, Gershon AS, et al. The effects of the health system response to the COVID-19 pandemic on chronic disease management: A narrative review. Risk Manag Healthc Policy 2021;14:575–584. 33623448 10.2147/RMHP.S293471 PMC7894869

[30] Heckert C. The bureaucratic violence of the health care system for pregnant immigrants on the United States-Mexico Border. Human Org 2020;79(1):33–42.

[31] Iness AN, Abaricia JO, Sawadogo W, et al. The effect of hospital visitor policies on patients, their visitors, and health care providers during the COVID-19 pandemic: A systematic review. Am J Med 2022;135(10):1158.e3–1167.e3. 35472383 10.1016/j.amjmed.2022.04.005 PMC9035621

[32] Guerra-Reyes L, Palacios I, Ferstead A. Managing precarity: Understanding Latinas' sexual and reproductive care-seeking in a Midwest emergent Latino community. Qual Health Res 2021;31(5):871–886. 33467985 10.1177/1049732320984430

[33] Shi L, Lebrun LA, Tsai J. The influence of English proficiency on access to care. Ethnicity Health 2009;14(6):625–642. 19953393 10.1080/13557850903248639

[34] Rosenberg E, Seller R, Leanza Y. Through interpreters' eyes: Comparing roles of professional and family interpreters. Patient Educ Couns 2008;70(1):87–93. 18031970 10.1016/j.pec.2007.09.015

[35] Hadziabdic E, Albin B, Heikkilä K, et al. Family members' experiences of the use of interpreters in healthcare. Prim Health Care Res Dev 2014;15(02):156–169. 23402584 10.1017/S1463423612000680

[36] AAFP. Telehealth and Telemedicine [Internet]. American Academy of Family Physicians. Available from: https://www.aafp.org/about/policies/all/telehealth-telemedicine.html [Last accessed: March 15, 2023].

[37] Eruchalu CN, Pichardo MS, Bharadwaj M, et al. The expanding digital divide: Digital health access inequities during the COVID-19 pandemic in New York City. J Urban Health 2021;98(2):183–186. 33471281 10.1007/s11524-020-00508-9 PMC7816740

[38] Gergen Barnett K, Mishuris RG, Williams CT, et al. Telehealth's double-edged sword: Bridging or perpetuating health inequities? J Gen Intern Med 2022;37(11):2845–2848. 35352272 10.1007/s11606-022-07481-w PMC8963395

[39] Merid B, Robles MC, Nallamothu BK. Digital redlining and cardiovascular innovation. Circulation 2021;144(12):913–915. 34543062 10.1161/CIRCULATIONAHA.121.056532

[40] Uiters E, Devillé W, Foets M, et al. Differences between immigrant and non-immigrant groups in the use of primary medical care; a systematic review. BMC Health Serv Res 2009;9(1):1–10. 19426567 10.1186/1472-6963-9-76 PMC2689181

